# Supplementary material for: Learning curve and surgical outcome of robotic assisted colorectal surgery with ERAS program
Source: Sci Rep. 2022 Nov 29;12:20566. doi: 10.1038/s41598-022-24665-w (PMC9709162; doi:10.1038/s41598-022-24665-w)
Supplement: Supplementary file 1 — Supplementary Information 1. [file 41598_2022_24665_MOESM1_ESM.zip › LSC Raw data-Ñ[▒K/087/H_ñw╝╨░O▒Kñσ.pdf]

正式摘要(電子簽章：000000195453357)		
W55 / W55 ☐ 英文標題(注意:無英文姓名-請補病人基本檔!)	☐【讀取】憑證	

臺中榮民總醫院

出院病歷摘要

轉入號： 轉出號：

[redacted]

(8)流水編號	
(9)入院日期	109/07/22 (2020)
(10)轉科日期	
(11)出院日期	109/07/27 (2020) 住院天數計 5 日

診斷

- 入院 :Ascending colon cancer
- 出院 :Ascending colon cancer s/p laparoscopic right hemicolectomy on 2020/7/23

主訴

- for scheduled operation

病史

- This 55 years old male patient denied special systemic disease. He recieved regular health examination on 2020/7/17. In colonoscope, ascending colon tumor was noted which pathology report showed malignancy. He denied changed bowel habbit, body weight loss. He came to our OPD for help. Abdominal CT showed focal wall thickening of A-colon about 3.8cm in size without obvious lymphadenopathy in retroperitoneum or pelvis or lesion in bilateral lower lung. Thus, he was admitted for surgical intervention.

體檢發現

-
  - General appearance :
    - easy looking , conscious clear , without cardiopulmonary distress
    - Vital signs自動帶入date 體溫(BT) 脈搏(P) 呼吸(R) 血壓(BP) 身高(BH) 體重(BW) BMI
      20200722 36.3℃ 86次/分 14次/分 124/88mmHg 175.2cm 78kg 25.4
    - Vital signs: B.P. 124/88 mmHg , P.R. 86 /min , R.R. 14 /min , B.T. 36.3 ℃
  - HEENT :
    - Eyes: no anemic conjunctiva, no icteric sclera, normal EOM
    - ENT: no otorrhea, no rhinorrhea, no erythematous mucosa, no injected tonsil, no deformity
  - Neck: supple, freely movable, trachea midline,no deformity, no engorged jugular vein , no palpable thyroid or lymph node enlargement
  - Chest:
    - Inspection: Grossly normal, symmetric and fully expansion, no accessory muscle used, no operation scar, no spider angiomata, no gynecomastia
    - Palpation: no palpable mass or nodule, no subcutaneous emphysema, no tenderness
    - Ausculation: clear breathing sound
  - Heart:
    - Palpation: no thrill, no heave
    - Percussion: normal heart size
    - Ausculation: regular heart beat, normal S1, S2, no gallop, no murmur
  - Extremities: No deformity, full ranged of motion without limitation, no clubbing finger or nail cyanosis, no pitting edema, no ulcer or pigmentation, no obvious skin lesion, no varicose vein, no bone, joint or muscle tenderness

  CRS CONDITION

  - Karnofsky performance status: (100) Normal, no complaints, no evidence of disease
  - PE of Abdomen:
  - __1. Inspection: intact
  - __2. Ausculation: normoactive
  - __3. Palapation: soft
  - __4. Percussion: -
  - CEA: 2.64 ng/ml

-
  - Imaging Studies:
  - Colonoscope report: -
  - Figures:
    1. colon & rectum

手術日期及方法

- 手術日期及方法20200723 Laparoscopic right colectomy and anastomosis

住院治療經過

- After admission, operation with laparoscopic right hemicolectomy was done on 7/23 smoothly as scheduled. We let him try water and liquid diet on POD1. Defecation was noted on POD2. Under the stable condition, he was discharged on 7/27.

合併症

- nil

一般檢查

- 累積報告 :

DATE	TIME	ALB	TP	BIL.T	BIL.D	ALKP	AST	ALT	LDH	CHOL	TG	HDL-C	LDL	UA	NH3	AMY
1090722	1956		7.8								70			6.4		

DATE	TIME	WBC	RBC	HGB	HCT	MCV	PLT	BLST	NEUT	BAND	SEG	LYM	MON	EOS	BAS
1090727	0919	8200		14.1	41.1		290		67.9			22.6	7.0	2.3	0.2

DATE	TIME	SP-GR	PH	PROT	GLUC	KETON	BILI	UROB	OB	NITR	LEU	RBC	WBC	EPCEL
1090726	1848	1.020	6.0	Negative	Negative	Trace	Negative	0.2	Negative	Negative	Trace	2-5	0-2	0-1

- 放射線報告 :檢驗項目 **:CHEST PA VIEW** 報告日期 **:1090722**

主　訴：
Result:
Finding:
> Normal heart size.
> No active lung lesions.
> Intact bony thorax.
Impression:
As above description.

檢驗項目 **:CT ABDOMEN-SURVEY** 報告日期 **:1090723**
主　訴：
Result:
Finding:
Imaging Report Form for Colorectal Carcinoma (AJCC 8th)
1. Imaging Date: 20200717
   Imaging Modality, Imaging by
   CT scan
   Imaging Protocol: CT of whole abdomen post IV and oral contrast enhancement
2. Tumor location / Size
   Location:
   Ascending
   Size:
   Measurable: 3.8 cm (largest diameter)
3. Tumor invasion
   T1/T2: Tumor within colonic or rectal wall
4. Regional nodal metastasis
   NX: Not assessable
5. Distant metastasis (In this study)
   No or Equivocal
6. Other findings: 1) Focal wall thickening of A-colon about 3.8cm in size, colon cancer may be compatible.
   2) Smaller than 0.7cm low density lesions in liver, hepatic cysts is first considered. Suggest F/U
   3) Bilateral renal cysts and a small left renal stone
   4) No abnormal focal lesion in GB, pancreas and spleen.
   5) No abnormal lymphadenopathy in retroperitoneum or pelvis.
   6) No abdnomal lesion in bilateral lower lung

Impression:
A-colon cancer

特殊檢查

病理報告

其它

出院時情況

- 3.改門診治療

出院指示

藥名	劑量	劑量單位	用法	頻次	數量	數量單位	現況	天數	單位
Mag. Oxide tab	2	TAB	PO	TID	60	TAB	TH	10	天
Ultracet tab	1	TAB	PO	Q6HPRN	28	TAB	TH	7	天
UroGEN tab 50mg	1	TAB	PO	TIDAC	30	TAB	TH	10	天

出院計劃

主治醫師 2062F 蔣鋒帆 / 住院醫師 2331F 陳凱祥

正式摘要(**電子簽章：000000195453357**)

W55 / W55

doctorRank:B
